# Oncogenic Actions of the Nuclear Receptor Corepressor (NCOR1) in a Mouse Model of Thyroid Cancer

**DOI:** 10.1371/journal.pone.0067954

**Published:** 2013-06-26

**Authors:** Laura Fozzatti, Jeong Won Park, Li Zhao, Mark C. Willingham, Sheue-yann Cheng

**Affiliations:** Laboratory of Molecular Biology, Center for Cancer Research, National Cancer Institute, National Institutes of Health, Bethesda, Maryland, United States of America; Roswell Park Cancer Institute, United States of America

## Abstract

Studies have suggested that the nuclear receptor corepressor 1 (NCOR1) could play an important role in human cancers. However, the detailed molecular mechanisms by which it functions *in vivo* to affect cancer progression are not clear. The present study elucidated the *in vivo* actions of NCOR1 in carcinogenesis using a mouse model (*Thrb^PV/PV^* mice) that spontaneously develops thyroid cancer. *Thrb^PV/PV^* mice harbor a dominantly negative thyroid hormone receptor β (TRβ) mutant (denoted as PV). We adopted the loss-of-the function approach by crossing *Thrb^PV^* mice with mice that globally express an NCOR1 mutant protein (NCOR1ΔID) in which the receptor interaction domains have been modified so that it cannot interact with the TRβ, or PV, in mice. Remarkably, expression of NCOR1ΔID protein reduced thyroid tumor growth, markedly delayed tumor progression, and prolonged survival of *Thrb^PV/PV^Ncor1* ***^ΔID/ΔID^*** mice. Tumor cell proliferation was inhibited by increased expression of cyclin-dependent kinase inhibitor 1 (p21^waf1/cip1^; *Cdkn1A*), and apoptosis was activated by elevated expression of pro-apoptotic BCL-Associated X (*Bax*). Further analyses showed that p53 was recruited to the p53-binding site on the proximal promoter of the *Cdkn1A* and the *Bax* gene as a co-repressor complex with PV/NCOR1/histone deacetylas-3 (HDAC-3), leading to repression of the *Cdkn1A* as well as the *Bax* gene in thyroids of *Thrb^PV/PV^* mice. In thyroids of *Thrb^PV/PV^Ncor1* ***^ΔID/ΔID^*** mice, the p53/PV complex could not recruit NCOR1ΔID and HDAC-3, leading to de-repression of both genes to inhibit cancer progression. The present studies provided direct evidence *in vivo* that NCOR1 could function as an oncogene via transcription regulation in a mouse model of thyroid cancer.

## Introduction

Thyroid hormone receptors (TRs) are critical in mediating the genomic actions of the thyroid hormone (T3) in growth, development, differentiation, and maintaining metabolic homeostasis. Two TR genes, α and β, located on two different chromosomes, encode three major T3 binding TR isoforms (α1, β1, and β2). Studies using genetically engineered mice showed that, *in vivo*, TR isoforms have common functions, but can also exert isoform-dependent actions in target tissues [1], [2]. The transcription activity of TR is regulated at multiple levels. In addition to the regulation by T3 and types of DNA binding elements in the promoters of target genes, the transcriptional activity of TR is fine-tuned by a host of nuclear coactivators and corepressors [3], [4]. In the absence of T3, the unliganded TR recruits the nuclear receptor corepressor 1 (NCOR1) and the nuclear receptor corepressor 2/silencing mediator for retinoid and thyroid hormone receptors (NCOR2/SMRT) for transcriptional repression. Binding of T3 leads to a conformational change in the TR that releases the NCOR1/NCOR2 complex and allows for the recruitment of a multiprotein coactivator complex for transcriptional activation [5].

The important regulatory role of NCOR1 in receptor actions is evident in its aberrant interaction with receptors underlying human diseases such as resistance to thyroid hormone (RTH) [6], [7] and lipodystrophic severe insulin resistance [8]. RTH is caused by mutations in the *THRB* gene [9], [10]. Mutations of the peroxisome proliferator-activated receptor γ (*PPARγ)* lead to lipodystrophic severe insulin resistance [8]. In addition, aberrant interaction of NCOR1/SMRT with the fused gene products of the retinoic acid receptor α gene (*RARα)* or with the promyelocytic leukemia gene (*PML*) (PML-RAR-α) or with the promyelocytic leukemia zinc finger gene (*PLZF*) (PLZF-RAR-α) results in blocking myeloid differentiation [11]. The involvement of NCOR1 in other human cancers such as breast and bladder cancers was demonstrated by a close association of NCOR1 abnormal expression with cancer development [12], [13], [14], [15]. Recent studies by The Cancer Genome Atlas (TCGA) Research Network also uncovered homozygous gene deletion of NCOR1 in some patients with colon and rectum adenocarcinoma [16], prostate adenocarcinoma [17], ovarian carcinoma [18] or liver hepatocellular carcinoma (unpublished-Provisional in TCGA data base). Moreover, nonsense mutations and splicing variants of *NCOR1* were also found in patients with breast cancer [19]. While these association studies raised the possibility that NCOR1 could act to affect cancer progression, direct evidence of its roles in carcinogenesis *in vivo* is still lacking.

The availability of a mouse model that spontaneously develops metastatic follicular thyroid cancer (FTC) provided us with a powerful tool to assess the role of NCOR1 in cancer development and progression. The *Thrb^PV/PV^* mouse harbors a knockin dominant negative mutation, known as PV, in the *Thrb* gene locus [20]. The PV mutation was identified in a patient suffering from resistance to thyroid hormone [21]. As *Thrb^PV/PV^* mice age, their thyroids undergo pathological changes from hyperplasia to capsular and vascular invasion, anaplasia, and eventual metastasis to the lung [22]. The pathological progression, route, and frequency of metastasis in *Thrb^PV/PV^* mice are similar to that in human FTC. Extensive molecular analyses of altered signaling pathways show that, as found in human FTC, *Thrb^PV/PV^* mice exhibit aberrant signaling pathways that include constitutive activation of phosphatidyl inositol 3-kinase (PI3K)/Akt [23], [24] and integrin–Src–MAPK signaling [25] and aberrant accumulation of the oncogenic pituitary tumor transforming gene protein (PTTG) [26], [27] and ß-catenin [28]. Thus, the *Thrb^PV/PV^* mouse model faithfully recapitulates the molecular aberrations found in human thyroid cancer and is indeed a preclinical mouse model of FTC.

In the present studies, we adopted the loss-of-the function approach by crossing *Thrb^PV^* with mice that globally express an NCOR1 mutant protein (NCOR1ΔID). In this NCOR1ΔID mutant protein, two most amino terminal receptor interaction domains termed RID 3 and RID2, are missing. This mutant cannot associate with TR, as RID3 is absolutely required for NCOR1-TR interaction [29], [30], [31]. Consistently, we also showed that NCOR1ΔID mutant protein cannot interact with TRβPV *in vivo* [32]. The lack of interaction of TRβPV with NCOR1ΔID results in the amelioration of symptoms of resistance to thyroid hormone in *Thrb^PV^Ncor1^ΔID/ΔID^* mice, indicative that NCOR1 regulates the dominant negative action of TRβ mutants *in vivo* [32].

The present studies show that the expression of NCOR1ΔID in the thyroid of *Thrb^PV/PV^* mice inhibited tumor growth, prolonged survival, and delayed cancer progression. Tumor cell proliferation was inhibited by the increased expression of cyclin-dependent kinase inhibitor 1 (p21/WAF1; *Cdkn1A*), and the apoptosis was induced by the activated expression of the Bcl-2–associated X protein (*BAX*). Both the *Cdkn1A* and the *BAX* genes are direct downstream target genes of the tumor suppressor, p53. Detailed molecular analyses showed that the lack of receptor interaction domain in NCOR1ΔID led to an inability of the p53/PV complex to recruit the NCOR1/histone deacetylase-3 repressor complex to the promoters of these target genes, resulting in increased repression of these genes to inhibit cancer progression. The present study provided direct evidence *in vivo* to demonstrate that the NCOR1 could function as an oncogene via transcription regulation in thyroid carcinogenesis in a mouse model.

## Materials and Methods

### Mouse Strains

The animal study was carried out according to the protocol approved by the National Cancer Institute Animal Care and Use Committee. Mice harboring the *Thrb^PV^* gene (*Thrb^PV^* mice) were prepared and genotyped as described earlier [20]. *Thrb^PV/PV^Ncor1^ΔID/ΔID^* mice were bred by first crossing *Thrb^+/+^Ncor1^ΔID/+^* mice [29] with *Thrb^PV/+^* mice and then by crossing *Thrb^PV/+^Ncor1^ΔID/ΔID^* mice with *Thrb^PV/+^Ncor1^ΔID/ΔID^* mice [32]. Mice with different genotypes used in the present study were intercrossed several generations, and littermates with a similar genetic background were used in all experiments. Mice were monitored until they became moribund and therefore euthanized. Thyroids and other tissues were collected from *Thrb^PV/PV^Ncor1^+/+^* mice and *Thrb^PVPV^Ncor1^ΔID/ΔID^* mice for weighing, histological analysis, and molecular and biochemical studies.

### RNA Isolation and Quantitative Real-Time RT-PCR

Total RNA was extracted from thyroids of mice using TRIzol (Invitrogen) according to the manufacturer’s instructions. Quantitative real-time RT-PCR was performed with a Quantitect SYBR Green RT-PCR kit (QIAGEN, Valencia, CA), according to the manufacturer’s instructions and using a LightCycler thermal cycler (Roche Diagnostics). Total RNA (200 ng) was used in RT-PCR determinations as described previously [33]. The specific primers were as follows: *mp21* forward, 5′-CGCCGCGGTGTCAGAGTC-3′ and reverse, 5′-GCAGCAGGGCAGAGGAAG-3′; *mBax* forward, ’-CCACCAGCTCTGAACAGATC-3′ and reverse, 5′-CAGCTTCTTGGTGGACGCAT-3′; *mp53* forward, 5′-AGAGACCGCCGTACAGAAGA-3′ and reverse, 5′-CTGTAGCATGGGCATCCTTT-3′.

### Western Blot Analysis and Co-immunoprecipitation

Nuclear extracts of thyroids were prepared as previously described [32]. The protein samples (25 µg) were analyzed by Western blot as described previously [28]. Anti-phosphorylated retinoblastoma protein (pRb) (Ser780, #9307) and PUMA (#7467) were from Cell Signaling Technologies (Beverly, MA) (1∶500 dilution), anti-cyclin D1 (sc-450), BAX (sc-7480), Rb (sc-50), p21 (sc-6246) and p27 (sc-1641) antibodies were purchased from Santa Cruz Biotechnology (Santa Cruz, CA) and used at a 1∶200 dilution. Anti-p53 antibodies (OP03) were from Calbiochem (1∶500 dilution). Band intensities were quantified by using NIH IMAGE software (ImageJ 1.34 s; Wayne Rasband, NIH).

For co-immunoprecipitation to show the physical interaction of p53 with PV, 0.5 mg of total thyroid extracts was first incubated overnight with rabbit anti-TR (Code 600-401-A96, Rockland) in Tris-buffered saline-0.6% NP-40 with protease inhibitors (Roche) at 4°C. The samples were then mixed with 20 µl protein A-agarose (Roche) at 4°C for 2 hours, and beads were washed five times with TBS-0.6% NP-40 containing protease inhibitors. Bound proteins were analyzed by Western blot analysis using antibodies for p53 (OP03; Millipore, Inc.,) at a 1∶500 dilution and anti-NCOR1 (PHQQ; 2 µg/mL) [32].

### Histological Analysis

Thyroid gland, lung, and heart were dissected, fixed in 10% neutral buffered formalin (Sigma-Aldrich, St. Louis, MO), and subsequently embedded in paraffin. Sections of 5-µm thickness were prepared and stained with hematoxylin and eosin (H&E). For each animal, single random sections through the thyroid, through the lung, and through the heart were examined.

### Immunohistochemistry

IHC was performed as previously described with some modification [25]. Formalin-fixed paraffin thyroid sections were deparaffinized, rehydrated, heated to 98°C in 0.05% citraconic anhydride, pH 7.4 (Sigma-Aldrich, St. Louis, MO), for 1 hour and then blocked for 1 hour in 2% normal goat serum at room temperature. After washing in phosphate-buffered saline, slides were incubated at 4°C overnight with Ki-67 primary antibody (1∶300 dilution; Thermo Scientific, Fremont, CA; #RB-9043-P0). After washing, slides were incubated with goat anti-rabbit secondary antibody for 1 hour at room temperature and rinsed in phosphate-buffered saline. Slides were then incubated in 3,3′-diaminobenzidene (DAB substrate kit for peroxidase; Vector Laboratories, Burlingame, CA; SK-4100), and after staining development, they were counterstained in Gill’s Hematoxylin, rinsed and mounted in Permount (Fisher Scientific, Pittsburgh, PA). The proliferative index was calculated as the percentage of Ki-67-positive nuclei to the total number of nuclei on the thyroid section. Counting was performed using National Institutes of Health (NIH) IMAGE software (ImageJ 1.34 s; Wayne Rasband, NIH, Bethesda, MD).

### Chromatin Immunoprecipitation (ChIP) Assay

The ChIP assay was carried out using chromatin DNA prepared from thyroid tumors (50 mg/assay) of *Thrb1^PV/PV^Ncor1^+/+^* mice and *Thrb1^PV/PV^Ncor1^ ΔID/ΔID^* mice, as described previously [34]. The immunoprecipitated-DNA in 50 µl of TE and quantitative PCR was performed with 7900HT Fast Real-Time PCR System (Applied Biosystems) using QuantiFast SYBR Green RT-PCR Kit (204154, QIAGEN). The enrichment in signals was calculated as immunoprecipitation signals versus whole cell lysate inputs. The fold changes of thyroid tumors of *Thrb1^PV/PV^Ncor1^+/+^* or *Thrb1^PV/PV^Ncor1^ΔID/ΔID^* ChIP were normalized by negative control (mouse IgG). The ChIP primers used were *Cdkn1A* forward, 5′- TTTCTATCAGCCCCAGAGGA-3′; and reverse, 5′- TCACCCCACAGCTGGTAGTT-3′ and *Bax* forward, 5′- GGGGCGCGCGGATCCATTCC-3′; and reverse, 5′- GCTTCTGATGGACAGGGGGC-3′.

### Statistical Analysis

All data are expressed as mean ± the SEM. Differences between groups were examined for statistical significance using Student’s *t* test with the use of GraphPad Prism 4.0a (GraphPad Software); p<0.05 is considered statistically significant.

## Results

### NCOR1ΔID Prolongs Survival and Delays Thyroid Carcinogenesis of *Thrb^PV/PV^* Mice

**Figure 1:**
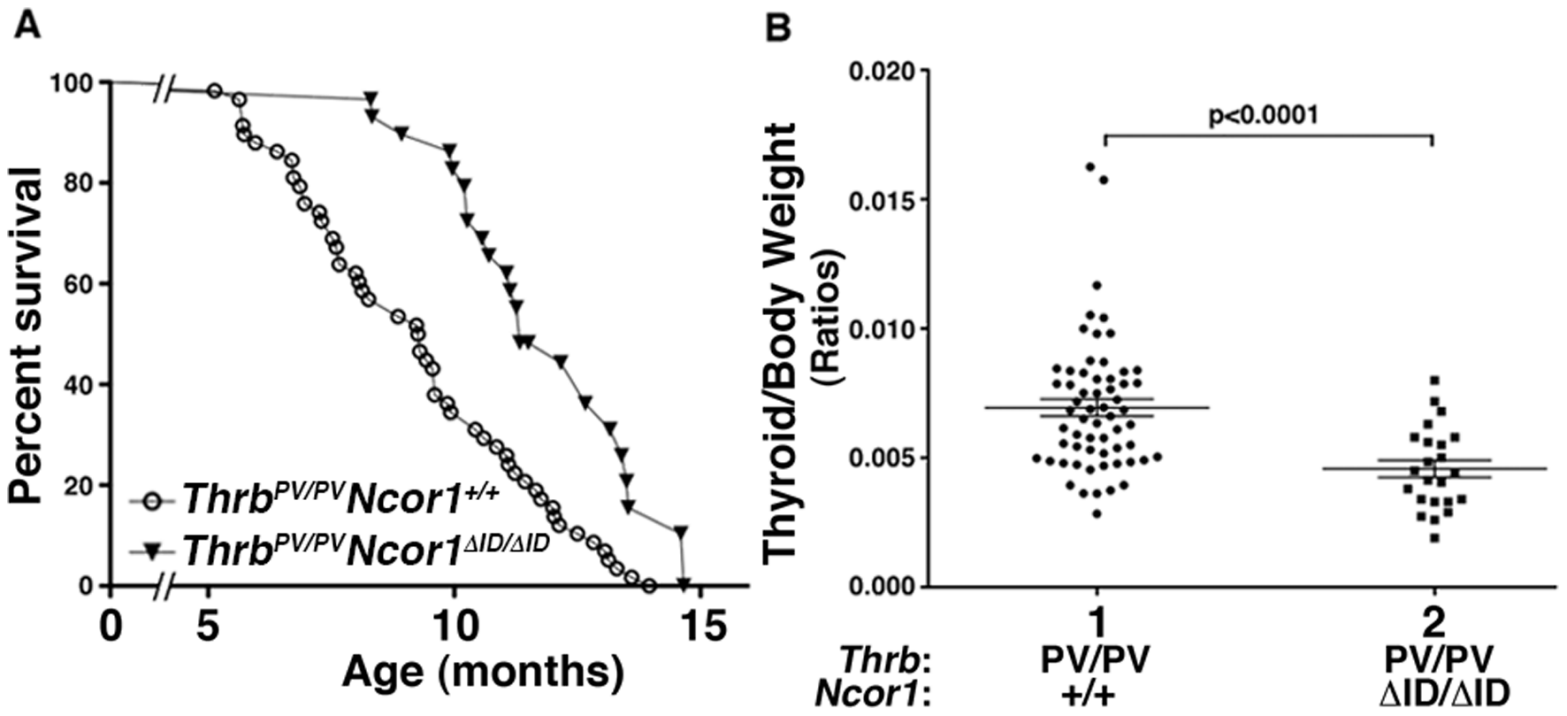
Expression of NCOR1ΔID increases survival of *Thrb^PV/PV^Ncor1^ΔID/ΔID^* mice. (**A**) Kaplan-Meier survival curves for *Thrb^PV/PV^Ncor1^+/+^* and *Thrb^PV/PV^Ncor1^ΔID/ΔID^* mice up 15 months of age. *Thrb^PV/PV^Ncor1^ΔID/ΔID^* (n = 29) survived significantly longer than *Thrb^PV/PV^Ncor1^+/+^* mice (n = 58) (*p*<0.0001). (**B**) Thyroids of *Thrb^PV/PV^Ncor1^+/+^* and *Thrb^PV/PV^Ncor1^ΔID/ΔID^* mice at the ages of 5–15 months were dissected and weighed. The data are presented as the ratios of thyroid weight to body weight. The differences between the thyroid weights of *Thrb^PV/PV^Ncor1^+/+^* and *Thrb^PV/PV^Ncor1^ΔID/ΔID^* mice were significant (*p*<0.0001), as determined by Student’s *t*-test analysis.

We have previously shown that *Thrb^PV/PV^* mice have high mortality caused by FTC [22]. We monitored the effects of the expression of NCOR1ΔID on thyroid carcinogenesis by first comparing the survival of *Thrb^PV/PV^Ncor1^ΔID/ΔID^* mice with that of *Thrb^PV/PV^Ncor1^+/+^* mice. Mice were monitored until they became moribund with signs of palpable tumors, rapid weight loss, hunched postures, and labored breathing. Figure 1A shows that *Thrb^PV/PV^Ncor1^ΔID/ΔID^* mice survived significantly longer (p<0.01; 50% survival age: 11.3 months, n = 29) than did *Thrb^PV/PV^Ncor1^+/+^* mice (50% survival age: 9.3 months, n = 58) during the 15-month observation period. Figure 1B shows that the expression of NCOR1ΔID led to a significant 35% reduction in thyroid weight in *Thrb^PV/PV^Ncor1^ΔID/ΔID^* mice (data set 2 vs. 1; p<0.0001).

**Figure 2:**
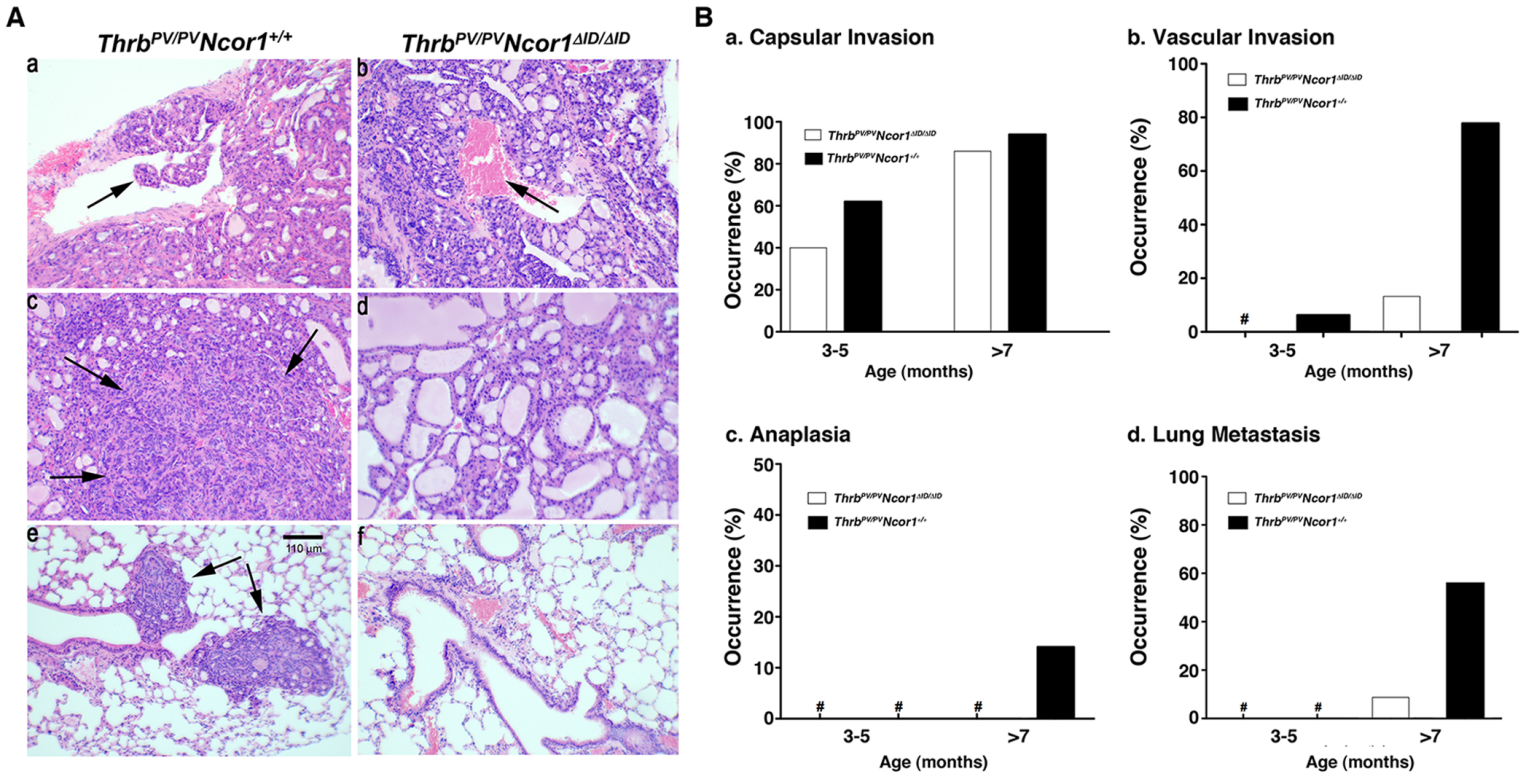
Expression of NCOR1ΔID delays thyroid cancer progression in *Thrb^PV/PV^Ncor1^ΔID/ΔID^* mice. (**A**) Hematoxylin and eosin (H&E) staining of thyroid sections (top and middle rows) and lung sections (bottom row) of *Thrb^PV/PV^Ncor1^+/+^* (a, c, and e) and *Thrb^PV/PV^Ncor1^ΔID/ΔID^* (b, d, and f) mice. Histological sections from tissues of *Thrb^PV/PV^Ncor1^+/+^* mice showed evidence of (a) vascular invasion in thyroid (arrow), (c) anaplasia in thyroid (arrows), and (e) metastatic lesions in lung (arrows). Sections of thyroids and lungs from *Thrb^PV/PV^Ncor1^ΔID/ΔID^* showed blood vessels (b) without vascular invasion (arrow), (d) without anaplasia, and (f) lung without metastatic lesions. (**B**) Comparison of age-dependent percentage occurrence of capsular invasion (a), vascular invasion (b), anaplasia (c), and lung metastasis (d). The data are expressed as the percentage of occurrence of total mutant mice examined. The symbol “#” indicates 0% occurrence.

The effect of NCOR1ΔID on the pathological progression was assessed by histopathological analysis as the *Thrb^PV/PV^Ncor1^ΔID/ΔID^* mice aged. Figure 2A shows representative pathological features of the thyroids and lungs from the 3- to 15-month-old *Thrb^PV/PV^Ncor1^+/+^* and *Thrb^PV/PV^Ncor1^ΔID/ΔID^* mice (left and right columns, respectively). In the thyroids of *Thrb^PV/PV^Ncor1^+/+^* mice at the age of 7 months, advanced vascular invasion and focal anaplasia were frequently observed (arrows in Figure 2Aa and 2Ac, respectively). Moreover, lung metastases (Figure 2Ae, arrows) were frequent in *Thrb^PV/PV^Ncor1^+/+^* mice. In *Thrb^PV/PV^Ncor1^ΔID/ΔID^* mice, however, vascular invasion was rarely observed (Figure 2Ab), but with no detectable anaplasia (Figure 2Ad) and a marked reduction in the occurrence of lung metastasis (Figure 2Af). These pathohistological observations are summarized in Figure 2B. At the younger age of 3–5 months, a lower occurrence of capsular invasion (∼20%) was observed for *Thrb^PV/PV^Ncor1^ΔID/ΔID^* mice than for *Thrb^PV/PV^Ncor1^+/+^* mice (Figure 2Ba), but a similar occurrence frequency of capsular invasion was detected for both mutant mice at older age (>7 months of age). No vascular invasion, anaplasia, or lung metastases were found in *Thrb^PV/PV^Ncor1^ΔID/ΔID^* mice (Figure 2Bb, c, and d) at younger ages of 3–5 months. For mice older than 7 months, the occurrence frequency of vascular invasion, anaplasia, and lung metastasis were 80%, 15%, and 60%, respectively, for *Thrb^PV/PV^Ncor1^+/+^* mice (Figure 2Bb, c, and d). However, the occurrence of vascular invasion and lung metastasis were 14% and 10%, respectively, for *Thrb^PV/PV^Ncor1^ΔID/ΔID^* mice, with no occurrence of anaplasia. Taken together, these results indicate that the expression of NCOR1ΔID delayed thyroid cancer progression and blocked loss of differentiation (anaplasia) in *Thrb^PV/PV^* mice.

### NCOR1ΔID Reduces Thyroid Growth by Inhibiting Cell Proliferation in *Thrb^PV/PV^Ncor1^ΔID/ΔID^* Mice

**Figure 3:**
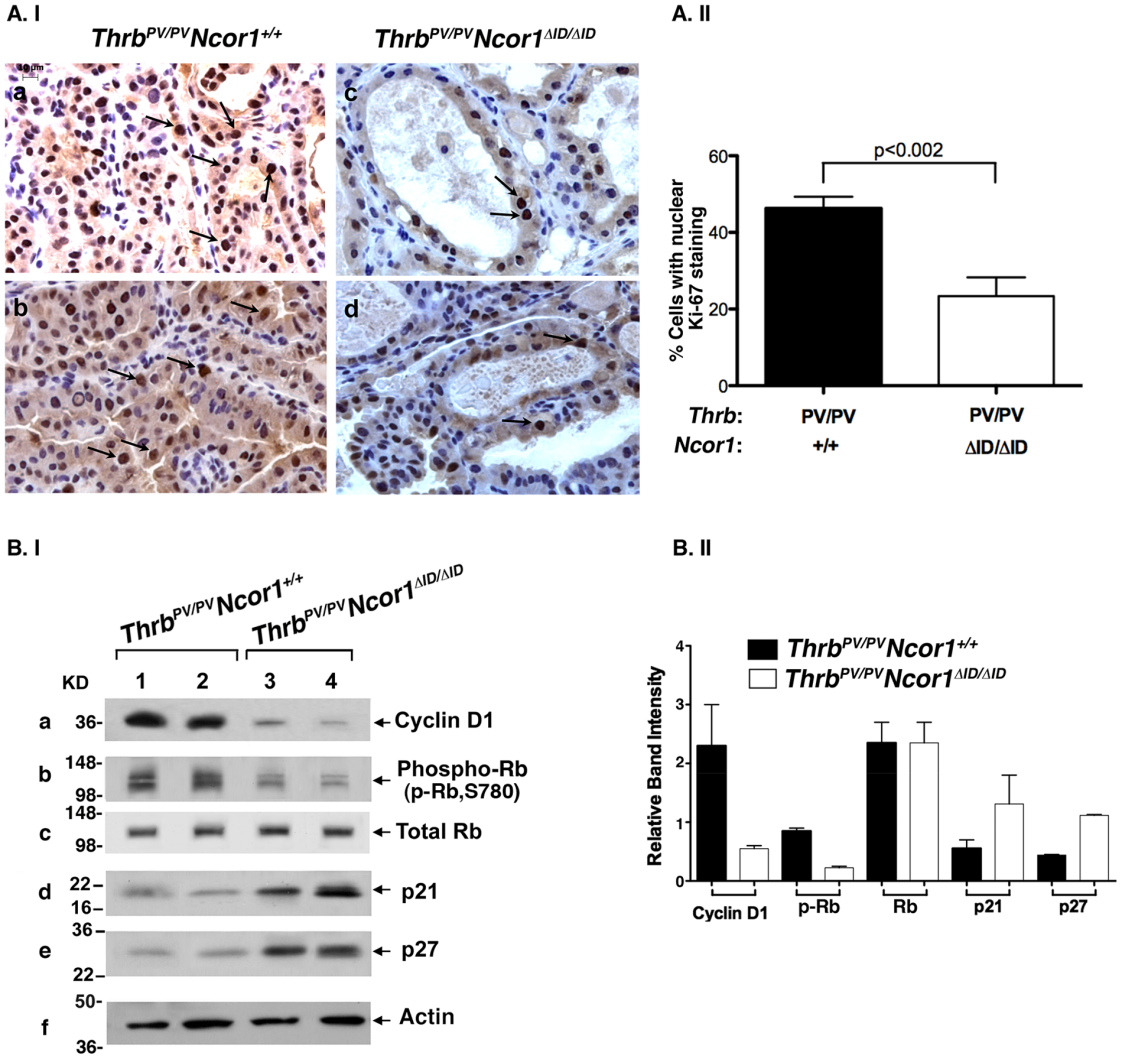
Expression of NCOR1ΔID inhibits proliferation of thyroid tumor cells of *Thrb^PV/PV^Ncor1^ΔID/ΔID^* mice. (**A.I**) Two representative microphotographs of stained Ki-67 on thyroid sections of *Thrb^PV/PV^Ncor1^+/+^* (a and b represent two different mice) and *Thrb^PV/PV^Ncor1^ΔID/ΔID^* mice (c and d represent two different mice). In the thyroids of *Thrb^PV/PV^Ncor1^ΔID/ΔID^* mice, fewer thyroid cells were stained with Ki-67 than in thyroids of *Thrb^PV/PV^Ncor1^+/+^* mice (arrows). (**A.II**) Positively nuclear Ki-67-stained thyroid cells from *Thrb^PV/PV^Ncor1^ΔID/ΔID^* and *Thrb^PV/PV^Ncor1^+/+^* mice were counted and expressed as percentage of total cells. The percentage of proliferating cells was significantly lower in the thyroids of *Thrb^PV/PV^Ncor1^ΔID/ΔID^* than *Thrb^PV/PV^Ncor1^+/+^* mice. (**B.I**) Nuclear protein extracts were prepared from thyroid tumors of *Thrb^PV/PV^Ncor1^+/+^* (lanes 1 and 2) or *Thrb^PV/PV^Ncor1^ΔID/ΔID^* (lanes 3 and 4) mice. Western blot analysis of cyclin D1, phosphorylated Rb, total Rb, p21, and p27 are as marked, and actin (panel f) was used as loading control. (**B.II**) Quantitation of band intensities shown in (B.I). The protein abundances of cyclin D1 and phosphorylated Rb were lower in the thyroids of *Thrb^PV/PV^Ncor1^ΔID/ΔID^* mice, whereas the protein abundance of p21 and p27 were higher in the thyroids of *Thrb^PV/PV^Ncor1^ΔID/ΔID^* mice.

The fact that thyroid growth was reduced in *Thrb^PV/PV^Ncor1^ΔID/ΔID^* mice (Figure 1B) prompted us to ask whether thyroid tumor cell proliferation was inhibited. We therefore examined the protein abundance of the nuclear proliferation marker, Ki-67, by immunohistochemical analysis. Figure 3A.I-a and -b show representative examples of intensive nuclear staining in thyroids of two *Thrb^PV/PV^Ncor1^+/+^* mice. In contrast, fewer nuclei were immuno-stained with Ki-67 proliferation marker in thyroids of *Thrb^PV/PV^Ncor1^ΔID/ΔID^* mice (Figure 3A.I-c and -d, n = 2). Cells with Ki-67 immuno-stained nuclei were counted and the quantitative data are shown in Figure 3A.II. The quantitative analysis shows that the number of thyroid cells with Ki-67 stained nuclei was 50% lower in *Thrb^PV/PV^Ncor1^ΔID/ΔID^* mice than in *Thrb^PV/PV^Ncor1^+/+^* mice, indicating decreased cell proliferation in the thyroid of *Thrb^PV/PV^Ncor1^ΔID/ΔID^* mice.

This finding was further supported by the biochemical analysis in which the protein abundance of a key cell cycle regulator, cyclin D1, was markedly lower in the thyroids of *Thrb^PV/PV^Ncor1^ΔID/ΔID^* mice (Figure 3B.I, panel a, lanes 3 & 4) than in *Thrb^PV/PV^Ncor1^+/+^* mice (lanes 1 & 2). Moreover, the reduced cyclin D1 led to a lower protein abundance of phosphorylated retinoblastoma protein (pRb) (Figure 3B.I, panel b, compare lanes 1 & 2 with lanes 3 & 4) without changing the total Rb protein levels (panel c). The reduction in the phosphorylated Rb impeded the progression of the cell cycle from the G1 to the S phase. Consistently, the protein abundance of cyclin-dependent kinase inhibitor p21 and p27 (Figure 3B.I, panels d and e, respectively) was also lower in the thyroids of *Thrb^PV/PV^Ncor1^ΔID/ΔID^* mice than in *Thrb^PV/PV^Ncor1^+/+^* mice (compare lanes 3–4 to lanes 1–2). The quantitative data of the band intensities are shown in Figure 3B.II. Collectively, these results indicate that the expression of the NCOR1ΔID led to inhibition of tumor cell proliferation, in part, by delaying the G1-S cell cycle progression.

**Figure 4:**
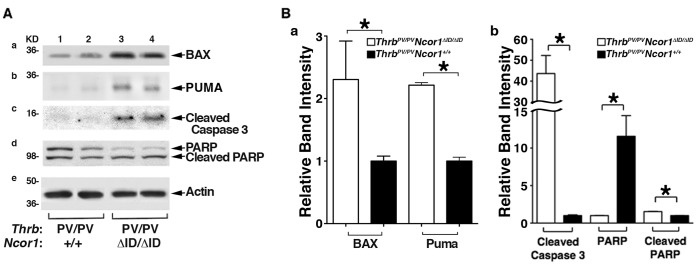
Expression of NCOR1ΔID promotes apoptosis of thyroid tumor cells of *Thrb^PV/PV^Ncor1^ΔID/ΔID^* mice. (**A**) Nuclear protein extracts were prepared from thyroid tumors of *Thrb^PV/PV^Ncor1^+/+^* (lanes 1 and 2) or *Thrb^PV/PV^Ncor1^ΔID/ΔID^* (lanes 3 and 4) mice. Shown are Western blot analyses of BAX (panel a), PUMA (panel b), cleaved caspase 3 (panel c), PARP and cleaved PARP (panel d), and actin loading control (panel e). (**B**) Quantitation of the band intensities shown in (A). *p<0.05 Protein abundance of BAX and PUMA (panel a), cleaved caspase 3 and cleaved PARP (panel b) was significantly higher and total PARP (panel b) was lower in the thyroids of *Thrb^PV/PV^Ncor1^ΔID/ΔID^* mice.

Whether apoptosis also contributed to decreased thyroid tumor growth shown in Figure 1B was also evaluated by examining the key regulators of apoptosis. The protein abundance of Bcl-2–associated X protein (BAX), which promotes apoptosis, was higher in thyroids of *Thrb^PV/PV^Ncor1^ΔID/ΔID^* mice than in *Thrb^PV/PV^Ncor1^+/+^* mice (Figure 4Aa, compare lanes 3 & 4 with 1 & 2). PUMA, which is a BH3 (Bcl-2 homology domain 3)-only protein that induces apoptosis through the mitochondria pathway, was also higher in thyroids of *Thrb^PV/PV^Ncor1^ΔID/ΔID^* mice than in *Thrb^PV/PV^Ncor1^+/+^* mice (Figure 4Ab, compare lanes 3 & 4 with 1 & 2). The quantitative band intensities are shown in Figure 4Ba, indicating a 2-fold increase in BAX and PUMA protein level. Furthermore, the increased cleaved caspase 3 (Figure 4A, panel b, lanes 3 & 4) and the decreased total poly ADP-ribose polymerase (PARP) but increased cleaved PARP (panel c, upper band and lower band, respectively, in lanes 3 & 4) in *Thrb^PV/PV^Ncor1^ΔID/ΔID^* mice as compared with *Thrb^PV/PV^Ncor1^+/+^* mice were indicative of the elevated apoptotic activity in thyroid tumor cells of *Thrb^PV/PV^Ncor1^ΔID/ΔID^* mice (also see quantitative data in Figure 4Bb). These results indicate that in addition to delaying the G1-S cell cycle progression, the increased apoptotic activity in thyroids of *Thrb^PV/PV^Ncor1^ΔID/ΔID^* mice also contributed to lower thyroid growth.

### Inhibition of Tumor Growth by Activation of the p53-signaling Pathway in the Thyroids of*Thrb^PV/PV^Ncor1^ΔID/ΔID^* Mice

**Figure 5:**
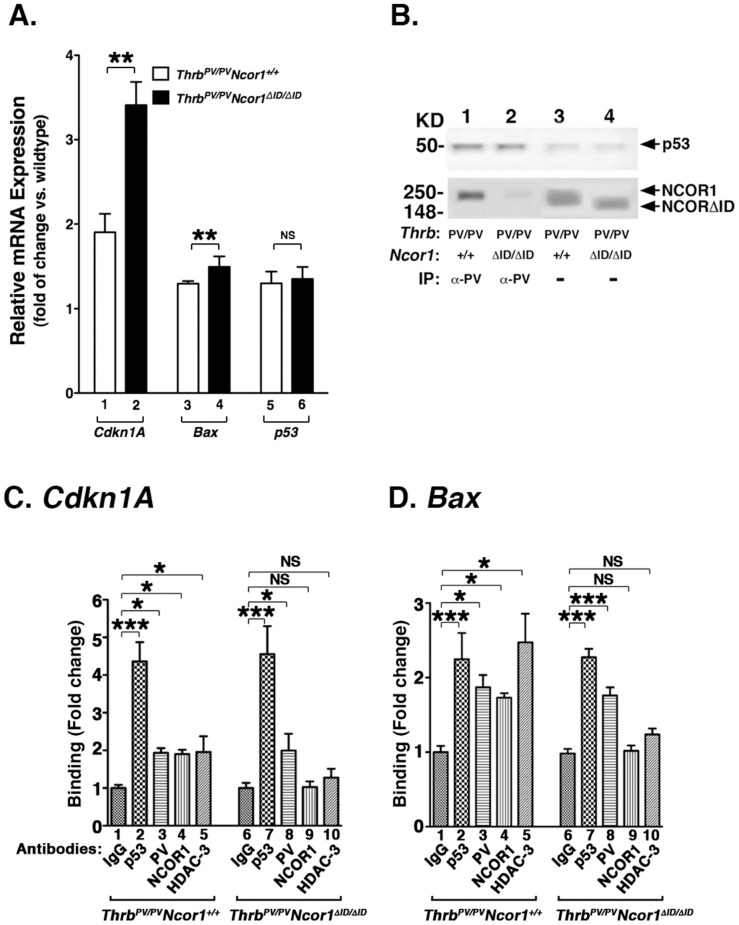
Expression of NCOR1ΔID alters expression of key regulators in cell proliferation and apoptosis in the thyroid of *Thrb^PV/PV^Ncor1^ΔID/ΔID^* mice. (A) Activated expression of the *Cdkn1A* and the *Bax* genes in the thyroid of *Thrb^PV/PV^Ncor1^ΔID/ΔID^* mice. Quantitative real-time RT-PCR was carried out as described in Methods and Materials. Each thyroid sample was run in triplicates with total mouse numbers of 3–5. The differences in the expression are significant in the expression of the *Cdkn1A* (lanes 1 and 2) and the *Bax* genes (lanes 3 and 4) between *Thrb^PV/PV^Ncor1^+/+^* and *Thrb^PV/PV^Ncor1^ΔID/ΔID^* mice (lanes 5 and 6). (B) Co-immunoprecipitation of PV with p53 and NCOR1 in the thyroid of *Thrb^PV/PV^Ncor1^+/+^* mice (lane 1), but not with NCOR1ΔID in thyroid extracts of *Thrb^PV/PV^Ncor1^ΔID/ΔID^* mice (lane 2). Lanes 3 and 4 show the p53 band (upper panel) and NCOR1 and NCOR1ΔID from direct Western blot analysis from the thyroid nuclear extracts of *Thrb^PV/PV^Ncor1^+/+^* and *Thrb^PV/PV^Ncor1^ΔID/ΔID^* mice, respectively. (C and D) Recruitment of PV and NCOR1 to the p53/DNA binding sites on the promoter of the *Cdkn1A* gene (C) and the *Bax* gene (D). ChIP assay was carried out using IgG (bars 1 and 6) or anti-p53 (bars 2 and 7) or anti-PV (bars 3 and 8) or anti-NCOR1 (bars 4 and 9) or anti-HDAC-3 (bars 5 and 10) antibodies as described in *Methods and Materials*. Binding was expressed as fold of changes in reference to the negative control in which mouse IgG was used in the immunoprecipitation.*p<0.05, and ***p<0.001. NS, not significant.

The findings that protein abundances of p21 and BAX were altered as shown above prompted us to examine whether their expression at the mRNA level was also affected. Indeed, we found that *Cdkn1A* (*p21^WAF1^*) mRNA levels were significantly higher in the thyroid of *Thrb^PV/PV^Ncor1^ΔID/ΔID^* mice than in *Thrb^PV/PV^Ncor1^+/+^* mice (Figure 5A, bar 2 vs. bar 1). Similarly, *Bax* mRNA level was higher in the thyroid of *Thrb^PV/PV^Ncor1^ΔID/ΔID^* mice than in *Thrb^PV/PV^Ncor1^+/+^* mice (Figure 5B, bar 4 vs. bar 3). These two genes are directly regulated by p53 [35], [36]. We therefore hypothesized that altering the activity of p53 in thyroids of *Thrb^PV/PV^Ncor1^ΔID/ΔID^* mice would lead to activation of transcription of the *Cdkn1A* and *Bax* genes. We first examined whether the expression of p53 mRNA and protein was altered, thereby changing the activity in the thyroid of *Thrb^PV/PV^Ncor1^ΔID/ΔID^* mice. We found that the expression of NCOR1ΔID had no effects of the expression of p53 at the mRNA level (bars 5 and 6, Figure 5A). In addition, we found that the protein abundance of p53 was similarly low in the thyroid of *Thrb^PV/PV^Ncor1^+/+^* and *Thrb^PV/PV^Ncor1^ΔID/ΔID^* mice (Figure 5B, upper panel, lanes 3 and 4, respectively). Therefore, the higher *Cdkn1A* and *Bax* mRNA levels were not due to activation of p53 transcription activity by the elevated p53 protein expression. Because it has been previously shown that wild-type TRs physically interact with p53 and negatively regulate the transcriptional activity of p53 [37], [38], we next explored the possibility that the activity of p53 could be altered via interaction with PV in thyroids of mutant mice. Co-immunoprecipitation analysis showed that PV was associated with p53 in the thyroid of *Thrb^PV/PV^Ncor1^+/+^* mice (Figure 5B, upper panel, lane 1). PV was also similarly associated with p53 in the thyroid of *Thrb^PV/PV^Ncor1^ΔID/ΔID^* mice (Figure 5B, upper panel, lane 2). Importantly, co-immunoprecipitation further showed that PV was also associated with NCOR1 in the thyroid nuclear extracts of *Thrb^PV/PV^Ncor1^+/+^* mice (Figure 5B, lower panel, lane 1), but did not interact with NCOR1ΔID in the thyroid nuclear extracts of *Thrb^PV/PV^Ncor1^ΔID/ΔID^* mice (lane 2). Lanes 3 and 4 show that a similar amount of NCOR1 and NCOR1ΔID, respectively, was detected by direct Western blot analysis (Figure 5B, lower panel). These data indicate that PV interacted with p53 and NCOR1 in a ternary complex (p53/PV/NCOR1) in the thyroid of *Thrb^PV/PV^Ncor1^+/+^* mice, but formed only a p53/PV complex in the thyroid of *Thrb^PV/PV^Ncor1^ΔID/ΔID^* mice.

**Figure 6:**
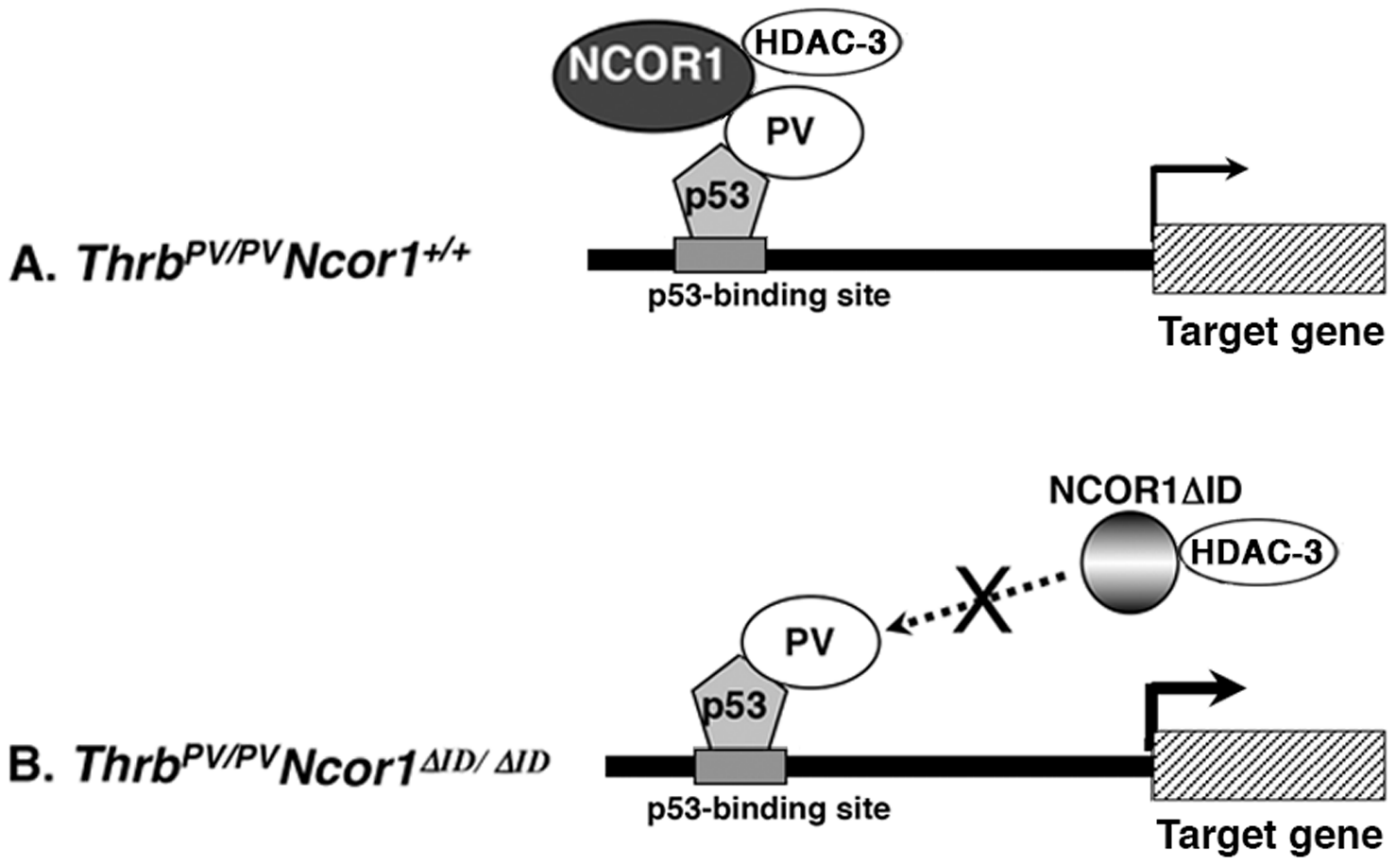
A proposed molecular model for the regulation of p53 downstream targets by PV. (**A**) Recruitment of the p53/PV/NCOR1/HDAC-3 repressor complex to the p53 binding site attenuates the expression of p53 target gene in the thyroid of *Thrb^PV/PV^Ncor1^+/+^* mice. (**B**) In the thyroid of *Thrb^PV/PV^Ncor1^ΔID/ΔID^* mice, the DNA-bound p53/PV complex cannot recruit NCOR1ΔID/HDAC-3 repressor complex, thereby alleviating the NCOR1/HDAC-3 repression effect, leading to activation of the p53-target gene.

The findings that *Cdkn1A* and *Bax* mRNA were higher in the thyroid of *Thrb^PV/PV^Ncor1^ΔID/ΔID^* than *Thrb^PV/PV^Ncor1^+/+^* mice (Figure 5A) provided us with a tool to further investigate the mechanisms by which NCOR1 regulated thyroid carcinogenesis. We therefore used a chromatin immunoprecipitation (ChIP) assay to understand how the transcriptional activity of p53 was activated in the thyroid of *Thrb^PV/PV^Ncor1^ΔID/ΔID^* mice to drive the expression of the *Cdkn1A* and *Bax* genes to inhibit tumor growth. Figure 5C shows that when anti-p53 antibodies were used in the ChIP assays, a strong signal above the background (bar 2 vs. bar 1) was detected to indicate that p53 was recruited to the p53 binding sites on the promoter region of the *Cdkn1A* gene (–1961 to –1942 bp; see Figure 6A) [35] in the thyroid of *Thrb^PV/PV^Ncor1^+/+^* mice (Figure 5C, bar 2). Importantly, when anti-PV antibodies were used in the ChIP, significant recruitment of PV to the promoter of the *Cdkn1A* gene was detected (Figure 5C, bar 3). Concurrently, using anti-NCOR1 and histone deacetylase-3 (HDAC-3) antibodies, we also found that NCOR1 and HDAC-3 were recruited to the p53/PV complexes (Figure 5C, bars 4 and 5, respectively). These results indicate that in thyroid tumors of *Thrb^PV/PV^Ncor1^+/+^* mice, the p53/PV/NCOR1/HDAC-3 repressor complex was recruited to the promoter of the *Cdkn1A* gene to repress the expression of the *Cdkn1A* gene. In contrast, in thyroids of *Thrb^PV/PV^Ncor1^ΔID/ΔID^* mice (Figure 5C), although p53 (bar 7) and PV (bar 8) were recruited to the p53 binding sites on the promoter of the *Cdkn1A* gene, no recruitment of NCOR1ΔID or HDAC-3 was found with the p53/PV complexes (Figure 5C, bars 9 and 10, respectively; see also Figure 6B). Similar ChIP analysis shows the significant recruitment of p53/PV/NCOR1/HDAC-3 to the p53 binding sites (nt -781 to -768) on the promoter of the *Bax* gene in the thyroids of *Thrb^PV/PV^Ncor1^+/+^* mice (Figure 5D; bars 2–5). But in the thyroids of *Thrb^PV/PV^Ncor1^ΔID/ΔID^* mice, only significant recruitment of p53/PV to the p53 binding sites on the *Bax* promoter was detected (Figure 5D, bars 7 and 8), but no recruitment of NCOR1ΔID or HDAC-3 to the promoter of the *Bax* promoter was observed (bars 9 and 10 vs. bar 6). These *in vivo* results indicate that in thyroids of *Thrb^PV/PV^Ncor1^ΔID/ΔID^* mice, the lack of interaction of PV with NCOR1ΔID reversed the repressive effect of NCOR1/HDAC-3. This, in turn, re-activated the p53 transcription activity to increase the expression of the *Cdkn1A* and *Bax* genes, thus delaying tumor progression by inhibiting tumor cell proliferation and activation of apoptosis.

## Discussion

Using a preclinical mouse model of thyroid cancer, we showed that the expression of NCOR1ΔID in the thyroid of *Thrb^PV/PV^* mice decreased tumor growth, delayed cancer progression, and increased survival. Analyses of key regulators underlying these reduced cancer phenotypes identified the increased expression of two p53 downstream direct target genes, the *Cdkn1A* and *Bax* genes. We further showed that p53 was associated with PV on the p53 binding sites of the promoters of the *Cdkn1A* and the *Bax* genes. However, NCOR1ΔID, lacking the receptor interaction domain, cannot be recruited by p53/PV to form the NCOR1ΔID/HDAC-3 associated repressor complex, leading to the activation of these two genes. The increased expression of these genes resulted in the decreased proliferation and increased apoptosis of tumor cells. These findings provided direct evidence to demonstrate that, *in vivo*, NCOR1 can function as an oncogene to drive thyroid cancer progression. One mechanism we uncovered is via transcription regulation by constitute association with p53/PV to recruit the NCOR1/HDAC associated repressor complex, which leads to aberrant chromatin acetylation around the promoters of the p53 target genes (see Figure 6A). The repression of p53 target genes that function as tumor suppressors is therefore attenuated, and leads to cancer progression. Such oncogenic roles of NCOR1 have been demonstrated in acute promyelocyte leukemia. A fusion between the retinoic acid receptor α (RARα) and either promyelocyte leukemic (PML) or promyelocyte leukemic zinc finger (PLZF) genes sustain NCOR1 interaction. Consequently, RARα-mediated cell differentiation is blocked because of a condensed chromatin structure around the promoters of the RARα target genes [11]. However, at present we cannot exclude the possibility that NCOR1 could act via other pathways in addition to p53 signaling. Such possibilities await future exploration.

The present studies also uncovered a critical role of NCOR1 in regulating the oncogenic actions of a TR mutant, PV, via the p53 network of signaling. Previously, we showed that PV physically interacts with the C-terminal SH domain of the p85 subunit of phosphatidylinositol 3-kinase (PI3K), resulting in the activation of PI3K-AKT signaling to promote thyroid carcinogenesis of *Thrb^PV/PV^* mice [23]. Subsequently, we discovered that NCOR1, when present in the cytoplasm, could compete with PV for binding to the same C-terminal SH domain of the p85 subunit, thereby attenuating the aberrant activation of PI3K-AKT signaling by PV [39]. The present studies showed that recruitment of nuclear NCOR1/HDAC-3 by DNA-bound p53/PV attenuated the expression of two p53 target genes, *Cdkn1A* and *Bax*, to promote tumor cell proliferation and to decrease apoptosis, respectively. Therefore, NCOR1 acts in dual modes on the oncogenic actions of PV via nuclear and extranuclear actions to affect thyroid carcinogenesis of *Thrb^PV/PV^* mice. However, it is reasonable to postulate that the nuclear action could be the predominant mode of action as NCOR1 is mainly located in the nucleus. Moreover, p53, as a central node of a complex network of signaling, when bound to DNA and associated with PV/NCOR1/HDAC-3 repressor complex, could broadly attenuate the expression of an array of tumor suppressors to promote carcinogenesis. This mode of action is exemplified by the regulation of the expression of the *Cdkn1A* and the *Bax* genes (see Figure 6). Identification of additional p53 target genes affected by the recruitment of the p53/PV/NCOR1/HDAC-3 repressor complex to the p53 binding sites would reveal the breadth of the p53 network extensively affecting thyroid carcinogenesis. However, such studies are for future investigation.

Moreover, we also cannot exclude the possibility that NCOR1ΔID could act via other pathways other than p53 signaling.

NCOR1 and the homolog NCOR2/SMRT are two intensively studied co-repressors [40], [41]. They both contain a conserved bipartite nuclear-receptor-interaction domain (RID) and three independent repressor domains [42]. These corepressors interact with nuclear receptors via the motif, termed the CoRNR box [43]. However, numerous biochemical studies have suggested that RAR preferentially recruits SMRT, whereas TR preferentially recruits NCOR1 [44], [45], mainly because of specific sequences on the motifs as well as a TR-specific interaction domain present in NCOR1, but not SMRT [44]. Accordingly, in the present study, we used the mutant *Ncor1^ΔID^* mice in which the TR-interaction domains were deleted, thus preventing interaction with TR or PV [30]. We also focused our molecular analysis on the recruitment of the PV/NCOR1/HDAC-3 repressor complex by p53 to the p53 binding sites of target genes (Figure 5). However, at present, we cannot exclude the possibility that p53/PV could also form a PV/SMRT/HDAC-3 repressor complex, thereby contributing to thyroid carcinogenesis of *Thrb^PV/PV^* mice by repression of tumor suppressors that are downstream targets of the p53-signaling. Currently, the potential oncogenic actions of SMRT have not been extensively investigated. Whether SMRT plays a role in thyroid carcinogenesis would be interesting to address in future studies.

The present results demonstrated the amelioration of the cancer phenotypes by the expression of NCOR1ΔID in the thyroid of *Thrb^PV/PV^* mice. However, the expression of NCOR1ΔID cannot completely block the development of thyroid cancer. These findings suggest that while activation of the p53-downstream tumor suppressors (e.g., the *Cdkn1A* and the *Bax* genes) by NCOR1ΔID is crucial, other p53-independent pathways driven by the oncogenic actions of PV are ongoing to continuously propel thyroid cancer progression. Previously, we found that PV, via extranuclear actions, aberrantly activates PI3K/AKT [23], [24] and integrin–Src–MAPK signaling [25] and abnormally accumulates the oncogenic pituitary tumor transforming gene protein (PTTG) [26], [27] and ß-catenin [28]. These preclinical findings clearly show that there are multiple oncogenic molecular pathways interconnected to drive the progression. Thus, it is important to consider using combined-drug modalities to concurrently target different pathways for effective treatment. We have previously shown that treatment of *Thrb^PV/PV^* mice with a PI3K inhibitor, LY294002, effectively delays thyroid tumor progression and inhibits distant metastasis [46]. But inhibition of the PI3K/AKT pathway alone is not sufficient to completely block cancer development and progression. The present studies suggest that inhibitors of histone deacetylases could be used together in conjunction with inhibitors of PI3K/AKT or integrin–Src–MAPK pathways. HDAC inhibitors have been shown to inhibit cell proliferation [47], [48], [49], [50] and in combination with all-trans-retinoic acid to increase re-differentiation of human thyroid cancer cell lines [51]. Completed Phase I and II clinical trials using various HDAC inhibitors have shown promise for treating radioiodine-refractory metastatic thyroid cancer [52]. Thus, treatment combining HDAC inhibitors with newer generations of inhibitors in the PI3K/AKT and integrin–Src–MAPK pathways could improve the efficacy offered by a single-drug treatment.

## References

[1] Forrest D, Vennstrom B (2000) Functions of thyroid hormone receptors in mice. Thyroid 10: 41–52. 10691312 10.1089/thy.2000.10.41

[2] Cheng SY (2005) Isoform-dependent actions of thyroid hormone nuclear receptors: lessons from knockin mutant mice. Steroids 70: 450–454. 15862829 10.1016/j.steroids.2005.02.003

[3] Cheng SY (2000) Multiple mechanisms for regulation of the transcriptional activity of thyroid hormone receptors. Rev Endocr Metab Disord 1: 9–18. 11704997 10.1023/a:1010052101214

[4] Hollenberg AN, Forrest D (2008) The thyroid and metabolism: the action continues. Cell Metab 8: 10–12. 18590688 10.1016/j.cmet.2008.06.008

[5] Glass CK, Rosenfeld MG (2000) The coregulator exchange in transcriptional functions of nuclear receptors. Genes Dev 14: 121–141. 10652267

[6] Love JD, Gooch JT, Nagy L, Chatterjee VK, Schwabe JW (2000) Transcriptional repression by nuclear receptors: mechanisms and role in disease. Biochem Soc Trans 28: 390–396. 10961926

[7] Privalsky ML (2004) The role of corepressors in transcriptional regulation by nuclear hormone receptors. Annu Rev Physiol 66: 315–360. 14977406 10.1146/annurev.physiol.66.032802.155556

[8] Agostini M, Schoenmakers E, Mitchell C, Szatmari I, Savage D, et al (2006) Non-DNA binding, dominant-negative, human PPARgamma mutations cause lipodystrophic insulin resistance. Cell Metab 4: 303–311. 17011503 10.1016/j.cmet.2006.09.003 PMC1821092

[9] Weiss RE, Refetoff S (2000) Resistance to thyroid hormone. Rev Endocr Metab Disord 1: 97–108. 11704998 10.1023/a:1010072605757

[10] Ferrara AM, Onigata K, Ercan O, Woodhead H, Weiss RE, et al (2012) Homozygous thyroid hormone receptor beta-gene mutations in resistance to thyroid hormone: three new cases and review of the literature. J Clin Endocrinol Metab 97: 1328–1336. 22319036 10.1210/jc.2011-2642 PMC3319181

[11] Lin RJ, Nagy L, Inoue S, Shao W, Miller WH Jr, et al (1998) Role of the histone deacetylase complex in acute promyelocytic leukaemia. Nature 391: 811–814. 9486654 10.1038/35895

[12] Girault I, Lerebours F, Amarir S, Tozlu S, Tubiana-Hulin M, et al (2003) Expression analysis of estrogen receptor alpha coregulators in breast carcinoma: evidence that NCOR1 expression is predictive of the response to tamoxifen. Clin Cancer Res 9: 1259–1266. 12684393

[13] Zhang Z, Yamashita H, Toyama T, Sugiura H, Ando Y, et al (2006) NCOR1 mRNA is an independent prognostic factor for breast cancer. Cancer Lett 237: 123–129. 16019133 10.1016/j.canlet.2005.05.046

[14] Abedin SA, Thorne JL, Battaglia S, Maguire O, Hornung LB, et al (2009) Elevated NCOR1 disrupts a network of dietary-sensing nuclear receptors in bladder cancer cells. Carcinogenesis 30: 449–456. 19126649 10.1093/carcin/bgp005 PMC2722152

[15] Banwell CM, MacCartney DP, Guy M, Miles AE, Uskokovic MR, et al (2006) Altered nuclear receptor corepressor expression attenuates vitamin D receptor signaling in breast cancer cells. Clin Cancer Res 12: 2004–2013. 16609009 10.1158/1078-0432.CCR-05-1218

[16] The Cancer Genome Atlas Network (2012) Comprehensive molecular characterization of human colon and rectal cancer. Nature 487: 330–337. 22810696 10.1038/nature11252 PMC3401966

[17] Taylor BS, Schultz N, Hieronymus H, Gopalan A, Xiao Y, et al (2010) Integrative genomic profiling of human prostate cancer. Cancer Cell 18: 11–22. 20579941 10.1016/j.ccr.2010.05.026 PMC3198787

[18] The Cancer Genome Atlas Network (2011) Integrated genomic analyses of ovarian carcinoma. Nature 474: 609–615. 21720365 10.1038/nature10166 PMC3163504

[19] The Cancer Genome Atlas Network (2012) Comprehensive molecular portraits of human breast tumours. Nature 490: 61–70. 23000897 10.1038/nature11412 PMC3465532

[20] Kaneshige M, Kaneshige K, Zhu X, Dace A, Garrett L, et al (2000) Mice with a targeted mutation in the thyroid hormone beta receptor gene exhibit impaired growth and resistance to thyroid hormone. Proc Natl Acad Sci U S A 97: 13209–13214. 11069286 10.1073/pnas.230285997 PMC27204

[21] Parrilla R, Mixson AJ, McPherson JA, McClaskey JH, Weintraub BD (1991) Characterization of seven novel mutations of the c-erbA beta gene in unrelated kindreds with generalized thyroid hormone resistance. Evidence for two “hot spot” regions of the ligand binding domain. J Clin Invest 88: 2123–2130. 1661299 10.1172/JCI115542 PMC295818

[22] Suzuki H, Willingham MC, Cheng SY (2002) Mice with a mutation in the thyroid hormone receptor beta gene spontaneously develop thyroid carcinoma: a mouse model of thyroid carcinogenesis. Thyroid 12: 963–969. 12490073 10.1089/105072502320908295

[23] Furuya F, Hanover JA, Cheng SY (2006) Activation of phosphatidylinositol 3-kinase signaling by a mutant thyroid hormone beta receptor. Proc Natl Acad Sci U S A 103: 1780–1785. 16446424 10.1073/pnas.0510849103 PMC1413672

[24] Ringel MD, Hayre N, Saito J, Saunier B, Schuppert F, et al (2001) Overexpression and overactivation of Akt in thyroid carcinoma. Cancer Res 61: 6105–6111. 11507060

[25] Lu C, Zhao L, Ying H, Willingham MC, Cheng SY (2010) Growth activation alone is not sufficient to cause metastatic thyroid cancer in a mouse model of follicular thyroid carcinoma. Endocrinology 151: 1929–1939. 20133453 10.1210/en.2009-1017 PMC2851190

[26] Ying H, Furuya F, Zhao L, Araki O, West BL, et al (2006) Aberrant accumulation of PTTG1 induced by a mutated thyroid hormone beta receptor inhibits mitotic progression. J Clin Invest 116: 2972–2984. 17039256 10.1172/JCI28598 PMC1592548

[27] Kim CS, Ying H, Willingham MC, Cheng SY (2007) The pituitary tumor-transforming gene promotes angiogenesis in a mouse model of follicular thyroid cancer. Carcinogenesis 28: 932–939. 17127711 10.1093/carcin/bgl231

[28] Guigon CJ, Zhao L, Lu C, Willingham MC, Cheng SY (2008) Regulation of beta-catenin by a novel nongenomic action of thyroid hormone beta receptor. Mol Cell Biol 28: 4598–4608. 18474620 10.1128/MCB.02192-07 PMC2447128

[29] Astapova I, Lee LJ, Morales C, Tauber S, Bilban M, et al (2008) The nuclear corepressor, NCoR, regulates thyroid hormone action in vivo. Proc Natl Acad Sci U S A 105: 19544–19549. 19052228 10.1073/pnas.0804604105 PMC2614797

[30] Astapova I, Vella KR, Ramadoss P, Holtz KA, Rodwin BA, et al (2011) The nuclear receptor corepressor (NCoR) controls thyroid hormone sensitivity and the set point of the hypothalamic-pituitary-thyroid axis. Mol Endocrinol 25: 212–224. 21239618 10.1210/me.2010-0462 PMC3386544

[31] Astapova I, Hollenberg AN (2013) The in vivo role of nuclear receptor corepressors in thyroid hormone action. Biochim Biophys Acta 1830: 3876–3881. 22801336 10.1016/j.bbagen.2012.07.001 PMC3529203

[32] Fozzatti L, Lu C, Kim DW, Park JW, Astapova I, et al (2011) Resistance to thyroid hormone is modulated in vivo by the nuclear receptor corepressor (NCOR1). Proc Natl Acad Sci U S A 108: 17462–17467. 21987803 10.1073/pnas.1107474108 PMC3198316

[33] Ying H, Suzuki H, Zhao L, Willingham MC, Meltzer P, et al (2003) Mutant thyroid hormone receptor beta represses the expression and transcriptional activity of peroxisome proliferator-activated receptor gamma during thyroid carcinogenesis. Cancer Res 63: 5274–5280. 14500358

[34] Fozzatti L, Lu C, Kim DW, Cheng SY (2011) Differential recruitment of nuclear coregulators directs the isoform-dependent action of mutant thyroid hormone receptors. Mol Endocrinol 25: 908–921. 21474540 10.1210/me.2010-0474 PMC3100599

[35] Macleod KF, Sherry N, Hannon G, Beach D, Tokino T, et al (1995) p53-dependent and independent expression of p21 during cell growth, differentiation, and DNA damage. Genes Dev 9: 935–944. 7774811 10.1101/gad.9.8.935

[36] Miyashita T, Reed JC (1995) Tumor suppressor p53 is a direct transcriptional activator of the human bax gene. Cell 80: 293–299. 7834749 10.1016/0092-8674(95)90412-3

[37] Qi JS, Desai-Yajnik V, Yuan Y, Samuels HH (1997) Constitutive activation of gene expression by thyroid hormone receptor results from reversal of p53-mediated repression. Mol Cell Biol 17: 7195–7207. 9372952 10.1128/mcb.17.12.7195 PMC232577

[38] Yap N, Yu CL, Cheng SY (1996) Modulation of the transcriptional activity of thyroid hormone receptors by the tumor suppressor p53. Proc Natl Acad Sci U S A 93: 4273–4277. 8633054 10.1073/pnas.93.9.4273 PMC39525

[39] Furuya F, Guigon CJ, Zhao L, Lu C, Hanover JA, et al (2007) Nuclear receptor corepressor is a novel regulator of phosphatidylinositol 3-kinase signaling. Mol Cell Biol 27: 6116–6126. 17606624 10.1128/MCB.00900-07 PMC1952145

[40] Horlein AJ, Naar AM, Heinzel T, Torchia J, Gloss B, et al (1995) Ligand-independent repression by the thyroid hormone receptor mediated by a nuclear receptor co-repressor. Nature 377: 397–404. 7566114 10.1038/377397a0

[41] Chen JD, Evans RM (1995) A transcriptional co-repressor that interacts with nuclear hormone receptors. Nature 377: 454–457. 7566127 10.1038/377454a0

[42] Jepsen K, Rosenfeld MG (2002) Biological roles and mechanistic actions of co-repressor complexes. J Cell Sci 115: 689–698. 11865025 10.1242/jcs.115.4.689

[43] Perissi V, Staszewski LM, McInerney EM, Kurokawa R, Krones A, et al (1999) Molecular determinants of nuclear receptor-corepressor interaction. Genes Dev 13: 3198–3208. 10617569 10.1101/gad.13.24.3198 PMC317209

[44] Cohen RN, Brzostek S, Kim B, Chorev M, Wondisford FE, et al (2001) The specificity of interactions between nuclear hormone receptors and corepressors is mediated by distinct amino acid sequences within the interacting domains. Mol Endocrinol 15: 1049–1061. 11435607 10.1210/mend.15.7.0669

[45] Makowski A, Brzostek S, Cohen RN, Hollenberg AN (2003) Determination of nuclear receptor corepressor interactions with the thyroid hormone receptor. Mol Endocrinol 17: 273–286. 12554754 10.1210/me.2002-0310

[46] Furuya F, Lu C, Willingham MC, Cheng SY (2007) Inhibition of phosphatidylinositol 3-kinase delays tumor progression and blocks metastatic spread in a mouse model of thyroid cancer. Carcinogenesis 28: 2451–2458. 17660507 10.1093/carcin/bgm174

[47] Altmann A, Eisenhut M, Bauder-Wust U, Markert A, Askoxylakis V, et al (2010) Therapy of thyroid carcinoma with the histone deacetylase inhibitor MS-275. Eur J Nucl Med Mol Imaging 37: 2286–2297. 20680269 10.1007/s00259-010-1573-3

[48] Catalano MG, Pugliese M, Poli R, Bosco O, Bertieri R, et al (2009) Effects of the histone deacetylase inhibitor valproic acid on the sensitivity of anaplastic thyroid cancer cell lines to imatinib. Oncol Rep 21: 515–521. 19148530

[49] Catalano MG, Poli R, Pugliese M, Fortunati N, Boccuzzi G (2007) Valproic acid enhances tubulin acetylation and apoptotic activity of paclitaxel on anaplastic thyroid cancer cell lines. Endocr Relat Cancer 14: 839–845. 17914112 10.1677/ERC-07-0096

[50] Catalano MG, Fortunati N, Pugliese M, Poli R, Bosco O, et al (2006) Valproic acid, a histone deacetylase inhibitor, enhances sensitivity to doxorubicin in anaplastic thyroid cancer cells. J Endocrinol 191: 465–472. 17088416 10.1677/joe.1.06970

[51] Yuan GB, Kuang AR, Fan Q, Yu LB, Mi YX (2010) Combined effects of all-trans-retinoic acid and trichostatin A on the induction of differentiation of thyroid carcinoma cells. Chin J Cancer 29: 379–384. 20346212 10.5732/cjc.009.10589

[52] Russo D, Damante G, Puxeddu E, Durante C, Filetti S (2011) Epigenetics of thyroid cancer and novel therapeutic targets. J Mol Endocrinol 46: R73–81. 21325372 10.1530/JME-10-0150

